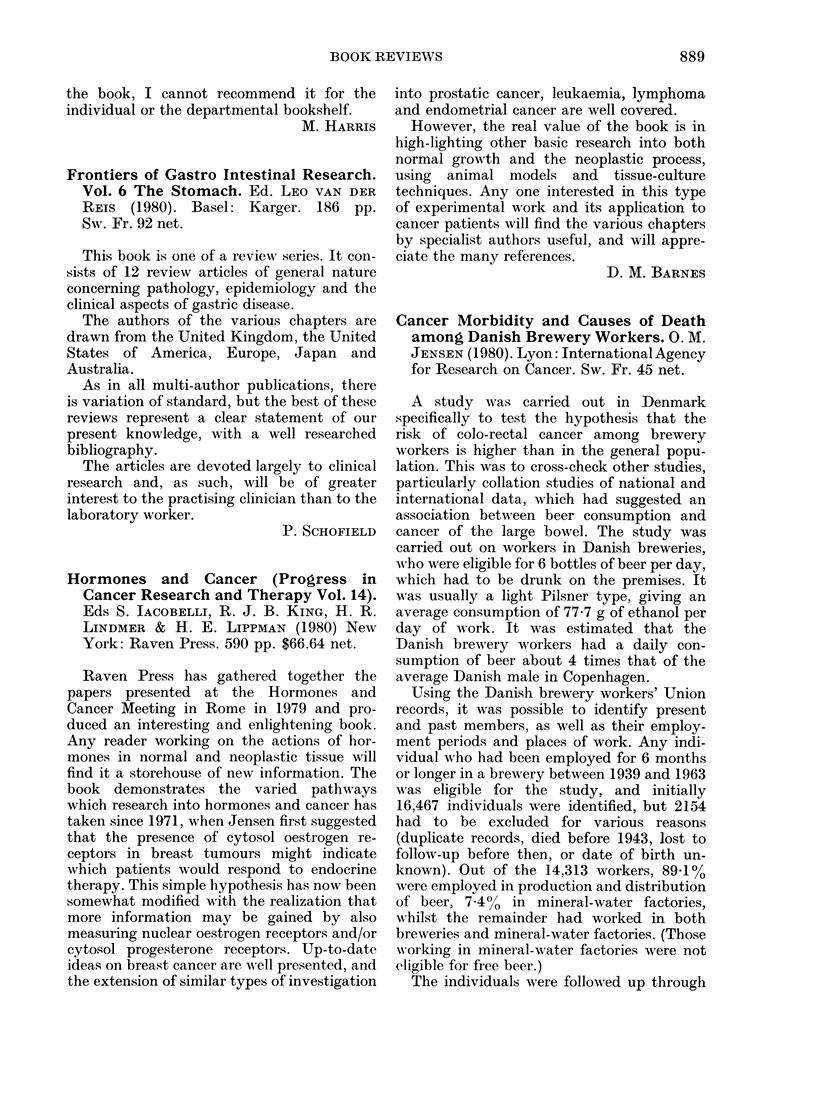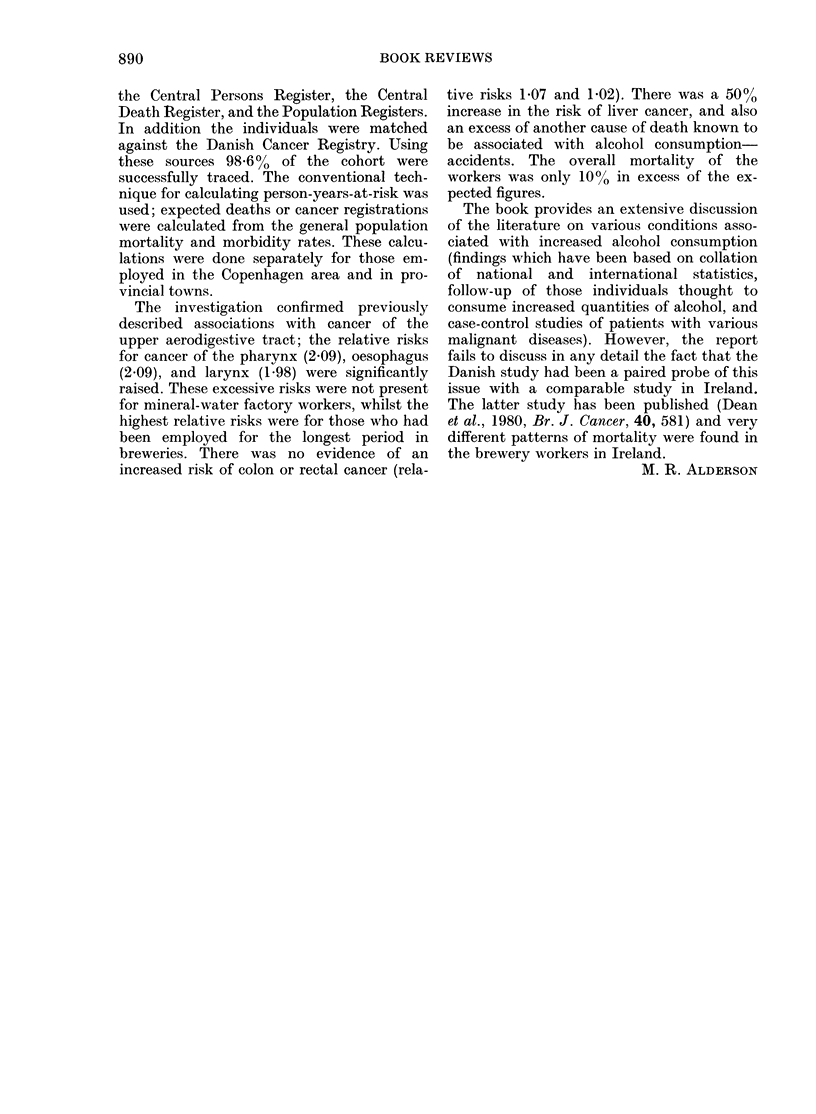# Cancer Morbidity and Causes of Death among Danish Brewery Workers

**Published:** 1981-06

**Authors:** M. R. Alderson


**Cancer Morbidity and Causes of Death among Danish Brewery Workers.** O. M. JENSEN (1980). Lyon: International Agency for Research on Cancer. Sw. Fr. 45 net.

A study was carried out in Denmark specifically to test the hypothesis that the risk of colo-rectal cancer among brewery workers is higher than in the general population. This was to cross-check other studies, particularly collation studies of national and international data, which had suggested an association between beer consumption and cancer of the large bowel. The study was carried out on workers in Danish breweries, who were eligible for 6 bottles of beer per day, which had to be drunk on the premises. It was usually a light Pilsner type, giving an average consumption of 77·7 g of ethanol per day of work. It was estimated that the Danish brewery workers had a daily consumption of beer about 4 times that of the average Danish male in Copenhagen.

Using the Danish brewery workers' Union records, it was possible to identify present and past members, as well as their employment periods and places of work. Any individual who had been employed for 6 months or longer in a brewery between 1939 and 1963 was eligible for the study, and initially 16,467 individuals were identified, but 2154 had to be excluded for various reasons (duplicate records, died before 1943, lost to follow-up before then, or date of birth unknown). Out of the 14,313 workers, 89·1% were employed in production and distribution of beer, 7·4% in mineral-water factories, whilst the remainder had worked in both breweries and mineral-water factories. (Those working in mineral-water factories were not eligible for free beer.)

The individuals were followed up through the Central Persons Register, the Central Death Register, and the Population Registers. In addition the individuals were matched against the Danish Cancer Registry. Using these sources 98·6% of the cohort were successfully traced. The conventional technique for calculating person-years-at-risk was used; expected deaths or cancer registrations were calculated from the general population mortality and morbidity rates. These calculations were done separately for those employed in the Copenhagen area and in provincial towns.

The investigation confirmed previously described associations with cancer of the upper aerodigestive tract; the relative risks for cancer of the pharynx (2·09), oesophagus (2·09), and larynx (1·98) were significantly raised. These excessive risks were not present for mineral-water factory workers, whilst the highest relative risks were for those who had been employed for the longest period in breweries. There was no evidence of an increased risk of colon or rectal cancer (relative risks 1·07 and 1·02). There was a 50% increase in the risk of liver cancer, and also an excess of another cause of death known to be associated with alcohol consumption—accidents. The overall mortality of the workers was only 10% in excess of the expected figures.

The book provides an extensive discussion of the literature on various conditions associated with increased alcohol consumption (findings which have been based on collation of national and international statistics, follow-up of those individuals thought to consume increased quantities of alcohol, and case-control studies of patients with various malignant diseases). However, the report fails to discuss in any detail the fact that the Danish study had been a paired probe of this issue with a comparable study in Ireland. The latter study has been published (Dean *et al.*, 1980, *Br. J. Cancer*, **40,** 581) and very different patterns of mortality were found in the brewery workers in Ireland.

M. R. Alderson